# The Origin of Salivary Calculus

**Published:** 1896-04

**Authors:** Henry M. Burchard

**Affiliations:** Philadelphia, Pa.


ARTICLE V.

### THE ORIGIN OF SALIVARY CALCULUS.

BY HENRY M. BURCHARD, D. D. S., M. D., PHILADELPHIA, PA.

(Continued from last number.)

A reaction which may have application here is the following: If to a solution of lactic acid milk of lime is added, a portion of the calcum is converted into calcium lactate; another portion is precipitated as calcium phosphate.

The proportion of lime-salts entangled appears to be small in this experiment. Upon filtering the solution, a coagulum (white) is left upon the filter-paper, which in drying assumes a dark color, a mixture of green and brown, about the color of some of the hard varieties of calculus.

To the filtrate add a solution of oxalic acid; a white precipitate separates and falls,—calcium oxalate.

As this is the reaction for calcium lactate, it is probable that there has been formed calcium lactate, soluble and left in solution, and calcium lacto-phosphate entangled in the coagulum.

It was experiment No. 1 which first drew the writer's attention to this matter as a possible solution to this undetermined question,—the *modus operandi* of calculus formation.

The conditions existing in the general cavity of the mouth (that is, the part containing the greater part of the saliva) are the reverse of the test-tube experiment. The fluid containing mucin and lime-salts was poured into a dilute solution of lactic acid. In a test-tube about two inches of a one per cent. solution of lactic acid were placed; there was then poured on it about one inch of saliva; immediately upon the contact of the fluids a coagulum formed,—formed so quickly that there was no apparent diffusion of the saliva into the lactic acid solution.

The more dilute the solutions of acid are used, the thinner are the coagula. With a one-half of one per cent. solution the coagulum is a viscid but well-defined mass floating in the general fluid. The coagulum forms so quickly that any particles suspended in the saliva are inevitably entangled in the meshes of the coagulum. In this event the calcium phosphate is probably combined with the lactic acid, forming a lacto-phosphate.

In the light of these investigations the writer recalls with pleasure a conversation between Dr. E. C. Kirk and himself over a year ago. In discussing the question of uratic deposits upon the roots of teeth, while one of these bodies was stewing in nitric acid, preparatory to making a "murexid" text, Dr. Kird said, "I believe all these calculary deposits will be found to belong to one great order. That salivary calculi will be found to be one group of several chemical bodies which are formed by the precipitation of lime-salts in colloid media, and this is the common factor in the formation of calculi in general. That about a nidus these substances will be deposited or formed in some definite manner; that they are more than mere agglomerations of lime-salts with extraneous matters; that they resemble calcoglobulin more than they do mere cemented precipitates, and I believe all calculi will have a family similarity in general structure, no matter in what part of the body they are formed"

It is purposed later to make sections of such teeth and calculi, prepared after the Weil method and try to view them by transmitted light, if possible. This was suggested to me by Dr. Kird during the conversation quoted. There is a field here for the specialist in histology.

Certain conditions are present, at least in part, which under other circumstances give origin to calcoglobulin; a lime-salt deposited in a colloid medium. I am not yet prepared to state that this is the result here; but the two factors mentioned are present.

"If a soluble salt of lime is mixed with a precipitant solution, the precipitate will be an amorphous powder.

"When the lime-salts are precipitated in gelatin, an albuminous, or other colloid medium, the character of the salt is materially altered. Instead of a powder there were found various curious but definite forms, quite unlike the character of crystals or powder formed without the intervention of the organic substance.

"If carbonate of lime be slowly formed in a thick solution of albumin, the resultant salt has changed its character. It is now in the form of globules laminated like tiny onions. These globules, when in contact, become agglomerated into a single laminated mass. The globular mass, at one time of mulberry-like form, loses the individuality of the constituent smaller globules and becomes smoothed down into a single mass or layer." (Rainey.)

"Mr. Rainey suggests as an explanation of the laminated structure that the smaller masses have accumulated in concentric layers which have subsequently coalesced.

"Professor Harting later found that other salts of lime would behave in a similar manner, and, by modifying the condition of the experiment, various forms might be produced.

"In the calcospherite, Mr. Rainey was aware that albumin actually entered into the composition of the globules, since it retained its form even after the application of an acid.

"Professor Harting has shown that the albumin left behind after treatment of a calcospherite with acid is no longer ordinary albumin; it is profoundly modified, and exceedingly resistant to the action of acids, alkalies, and boiling water. He proposes for the modified albumin the name of calcoglobulin, as the lime appears to be held in chemical combination, for the last traces of lime are retained very obstinately when calcoglobulin is submitted to the action of acids." (R. R. Andrews, after Rainey and Harting.)

The centers of lactic fermentation in the mouth are scattered in every mass of food-debris; the process is inactive, so the saliva is becoming charged with lactic acid at many places, hence the coagula would be scattered generally through the saliva.

The experiment noted has been repeated several times; the results are always the same. Chemically, the conditions or factors are analogous to those existing in the human mouth, addition of saliva being made to a solution of lactic acid. It is fair, therefore, to assume that in the mouth, when the saliva comes in contact with solutions of lactic acid, these coagula will form.

In the box-like sublingual cavity it is seen that its highest point is at the linguo cervical aspects of the inferior incisors, hence as the coagula are lighter than the liquid in which they are suspended, they would agglomerate at that situation, and by the movement of the tip of the tongue be pressed between the teeth, filling the cervical spaces, and into the depression or fold of the gingivæ.

In the folds, the recession or depression marking the junction of the gingivæ with the teeth, accumulations of debris are found. Through the mechanical cleansing by the tongue most of the material has been removed, but there remains a line of deposit which forms a food-supply to micro-organisms, and producing, it may be, substances which act as local irritants. It will be remembered that, as stated, in this fold of gum there open the mouths of numerous glands secreting mucin. In addition to the normal secretion at these points, the local irritation, due to the presence of foreign and irritating bodies and substances, will produce an increased and, it may be, an altered secretion in the glands.

There is another prolific source for such disturbance, —an acquired predisposition to gingivitis, or, it may be, stomatitis. From their structure the gums are adapted for rough usage; are fitted (and, no doubt, in all animals designed) to receive without injury a marked degree of pressure or buffeting during mastication. In addition to the cooking of food, rendering it a more fitting soil for the breeding of micro-organisms, rendering it more decomposable, the softening cf food-stuffs by the cooking process literally robs the gum-tissue and the supporting structures of the teeth of proper exercise. This lessening of functional activits (disuse) is a source of debility, so that an oral condition is engendered furnishing a lessened resistance to disease-producing agencies. The secretion containing mucin is exuding into a collection of fermenting material; mucin is coming in contact with lactic acid, and bathed in a fluid containing salts of calcium.

The pressure of the elastic gum upon the collection flattens the coagulum, and through the movements of the tongue or any muscular parts, the portion of the coagulum above the gum-margin is brushed away. The swollen gum-tissue forms a pocket, in which a coagulum of mucin containing lime-salts is protected from mechanical dislodgment, and thus, like any similar deposit, hardens with age. As is duly noted, the calculi ultimately cause the detachment of the gum from the tooth, and exhibit the congestive gum-margins characteristic of this condition. By the pressure to which the coagulum is subjected it is condensed, has much of its fluid pressed out. Artificial coagula are seen to acquire a dark tinge during a progressive drying, and resemble in appearance those subgingival deposits.

As will be inferred from this presentation, the writer is disposed to attribute a different origin (at least different in part) to the subgingival deposits, from that of the epigingival, those at the cervices of the inferior incisors and upper molars.

The question of the condensation, the agglomeration of the coagula at the cervices of the inferior incisors, appears to be due to the specific gravity of the coagula being less than that of the saliva. This being the highest point of a cavity, they accumulate there. No doubt after the formation of the coagula, these latter offer, by their physical character, a nidus for the deposition of calcium salts, and successive additions of coagula. Situated as they are opposite the openings of the salivary ducts, they have entangled in their substance the precipitated calcium phosphate.

Another probable direct course of additions to the concretions is that, as stated in the analysis of the saliva, it contains an amount of free carbon dioxid, enough to hold calcium salts in solution, both the phosphate and the small amount of carbonate present; upon exposure to the air the carbon dioxid escapes, and the calcium salt precipitates.

Successive coagula and lime deposits occur, massed between the teeth by the action of the tongue.

The next situation, the buccal faces of the superior molars. These are usually the softest of all deposits; this condition due no doubt to the absence of mucin in the secretion of the parotid gland. Mucin, however, is contained in the buccal pockets, even if only that secreted by the gingival and buccal glands. The calcium salts are precipitated, owing to the escape of carbonic acid, and are entangled in the coagula. As a rule these deposits are mechanically displaced by the action of the buccal muscles and food masses during mastication; they are always subjected to a free washing by the watery parotid secretion. If the masticatory function be lost or disused upon one side, there is an absence of the mechanical cleansing, and deposits of calculi occur more frequently. It has always been noted that these deposits were protective against caries to the surfaces covered by them. It is evident, therefore, that lactic acid cannot be present upon their under-surfaces; no doubt it has combined with the phosphate, forming a lacto-phosphate of calcium.

The most favorable conditions for the formation of calculi appear to be a cavity or space, in which the fluids of the mouth may rest as in a pool, and to have some rough projection to engage and retain precipitated matters; and, again, the presence of some foreign body which may act as a nidus to the agglomerating mass.

Accepting the truth of this hypothesis of the origin of calculi, it is evident that their prevention is essentially the prevention of the formation of lactic acid. It will be seen also that the same therapeusis is the prevention of dental caries.

The active cause of the production of these organisms is always present (the micro-organisms), and it is accepted that the condition of the mucous membrane and its glands determines in a great measure the amount and activity of these bodies. As the first means of prevention, the hygienic condition of the gums must form the first element. It is probable, as stated, that the debility of the soft tissues of the arches is due in great part to insufficient usage.

The second factor, the removal of fermentable material, as the process of cooking food, leaves an increased amount and adhesiveness of debris; mechanical dislodgment and removal are necessary. These means lessen the food-supply, the soil for the micro organisms; but as it is impracticable to remove it in toto, the next solution of the problem is the destruction of the organisms themselves,—this by means of antiseptics.

If the hypothesis presented is accepted, a fresh significance is given the use of antiseptic mouth-washes.

Among animals which are fed upon pasty food, particularly about the teeth of lap-dogs, pet terriers, etc., it is not unusual to find extensive deposits of calculi. These are always associated with more or less stomatitis, in part unquestionably due to the irritation produced by the calculi, and in some cases an oral catarrh has clearly preceded the deposits. I remember one case, a Blenheim terrier, subjected to the most miserable form of coddling, ill-feeding, and lack of exercise, whose teeth were covered by incrustation of hard, green deposits, and in addition there was a well-marked pyorrhea. I extracted several of the teeth for this animal, An old *Felis concolor* (puma), at the Philadelphia Zoological Garden, had a similar affection. Dark deposits could be seen along the necks of the teeth, the gums turged and showing a blue line over the deposits; several teeth were loosening.

The hypothesis as at present formulated is that salivary calculi are due to the action of lactic acid upon the secretions of the salivary glands and the glands of the oral mucous membrane. That by the union of these two substances a coagulum of mucin is formed, in which are deposited and entangled precipitated lime-salts, mainly lactophosphate. That the deposits are similar to calculi in other parts; but whether the union of the colloid with the lime-salts is more definite than a mixture is undetermined.

This paper is simply a statement of the results of experiments to date; it is purposed to do further work in this field, and these views as present expressed may be modified, but I believe the central idea will remain intact. It is presented as it is at this time at the request of the editor of the *Dental Cosmos*, and thus represents work in process of transition, not completeness.—*Dental Cosmos.*

